# Antisense oligonucleotide therapy corrects splicing in the common Stargardt disease type 1-causing variant *ABCA4* c.5461-10T>C

**DOI:** 10.1016/j.omtn.2023.02.020

**Published:** 2023-02-18

**Authors:** Melita Kaltak, Petra de Bruijn, Davide Piccolo, Sang-Eun Lee, Kalyan Dulla, Thomas Hoogenboezem, Wouter Beumer, Andrew R. Webster, Rob W.J. Collin, Michael E. Cheetham, Gerard Platenburg, Jim Swildens

**Affiliations:** 1 ProQR Therapeutics, Zernikedreef 9, 2333 CK Leiden, the Netherlands; 2 Department of Human Genetics, Radboud University Medical Center, Geert Grooteplein-Zuid 10, 6525 GA Nijmegen, the Netherlands; 3 Academic Alliance Genetics, Radboud University Medical Center, Geert Grooteplein-Zuid 10, 6525 GA Nijmegen, and Maastricht University Medical Center+, P. Debyelaan 25, 6229 HX Maastricht, the Netherlands; 4 UCL, Institute of Ophthalmology, 11-43 Bath Street, EC1V 9EL London, UK; 5 Moorfields Eye Hospital, 162 City Road, EC1V 2PD London, UK

**Keywords:** MT: Oligonucleotides: Therapies and Applications, RNA therapy, antisense oligonucleotides, Stargardt disease, splicing correction, retinal organoids, *ABCA4*

## Abstract

Stargardt disease type 1 (STGD1) is the most common hereditary form of maculopathy and remains untreatable. STGD1 is caused by biallelic variants in the *ABCA4* gene, which encodes the ATP-binding cassette (type 4) protein (ABCA4) that clears toxic byproducts of the visual cycle. The c.5461-10T>C p.[Thr1821Aspfs∗6,Thr1821Valfs∗13] variant is the most common severe disease-associated variant, and leads to exon skipping and out-of-frame *ABCA4* transcripts that prevent translation of functional ABCA4 protein. Homozygous individuals typically display early onset STGD1 and are legally blind by early adulthood. Here, we applied antisense oligonucleotides (AONs) to promote exon inclusion and restore wild-type RNA splicing of *ABCA4* c.5461-10T>C. The effect of AONs was first investigated *in vitro* using an *ABCA4* midigene model. Subsequently, the best performing AONs were administered to homozygous c.5461-10T>C 3D human retinal organoids. Isoform-specific digital polymerase chain reaction revealed a significant increase in correctly spliced transcripts after treatment with the lead AON, QR-1011, up to 53% correct transcripts at a 3 μM dose. Furthermore, western blot and immunohistochemistry analyses identified restoration of ABCA4 protein after treatment. Collectively, we identified QR-1011 as a potent splice-correcting AON and a possible therapeutic intervention for patients harboring the severe *ABCA4* c.5461-10T>C variant.

## Graphical abstract

**Figure:**
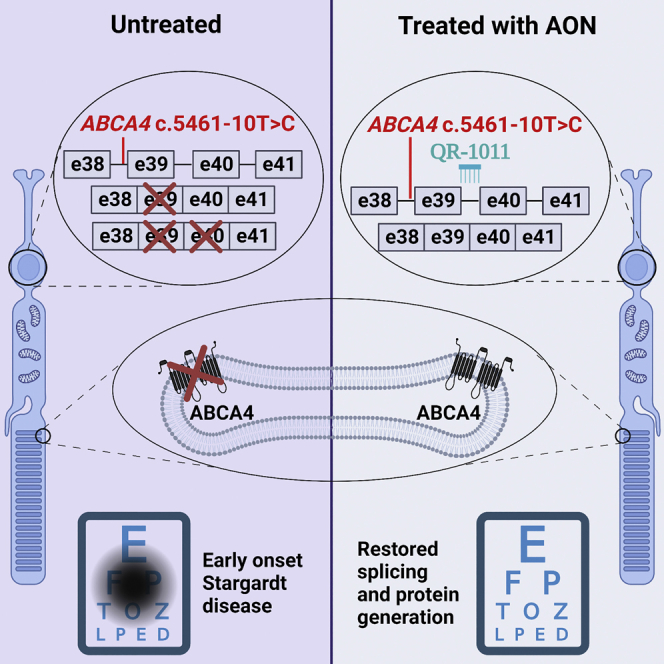

## Teaser

*ABCA4* c.5461-10T>C is the most common severe variant underlying Stargardt disease. It causes exon skipping, leading to loss of the ABCA4 protein, and there is no available treatment. Here, Kaltak et al. develop an antisense oligonucleotide-based therapy that rescued splicing and restored protein to clinically relevant levels in human retinal organoids.

## Introduction

The clearance of toxic retinoid metabolites from the visual cycle is essential for the maintenance of functional retinal pigment epithelium (RPE), as well as cone and rod photoreceptor cells in the retina. This clearance is in part carried out by the ATP-binding cassette, sub-family A, member 4 (ABCA4) protein, which is localized in the outer segment disk rims of photoreceptor cells. In the absence of functional ABCA4, N-retinylidene-phosphatidylethanolamine and the lipofuscin fluorophore A2E accumulate in the RPE, leading to the death of the RPE layer and the photoreceptor cells.^1^^,^^2^^,^^3^^,^^4^ In the case of two *ABCA4* null-alleles, disease progression is rapid and clinically recognized as autosomal recessive cone-rod dystrophy, whereas the involvement of alleles with residual ABCA4 function gives rise to Stargardt disease type 1 (STGD1).^5^^,^^6^^,^^7^ Although STGD1 is the most common form of inherited macular dystrophy, no treatment options are available, highlighting the importance of the development of therapeutic strategies. Studies on large cohorts of STGD1 patients have identified more than 2,200 disease-associated variants in *ABCA4*,^8^ the majority of which are missense variants, followed by mutations that alter pre-mRNA splicing.^9^^,^^10^^,^^11^^,^^12^

*ABCA4* c.5461-10T>C p.[Thr1821Aspfs∗6,Thr1821Valfs∗13] is a non-canonical splice site variant that causes the skipping of either exon 39, or exons 39 and 40 together, resulting in the production of out-of-frame *ABCA4* isoforms.^6^^,^^7^^,^^13^ It is the most common severe STGD1-causing variant.^8^^,^^14^^,^^15^^,^^16^ The onset of the first symptoms of STGD1 disease in individuals homozygous for the *ABCA4* c.5461-10T>C variant is often in the first decade of life, after which the progress of the disease can sharply accelerate and lead to legal blindness between the ages of 20 and 30.^7^^,^^17^ The steep deterioration of visual acuity in homozygous individuals is explained by dramatically decreased levels of wild-type ABCA4 protein.^7^^,^^13^^,^^18^

The eye is an isolated and, consequently, immune-privileged organ, which makes it an attractive target for the development of genetic therapies; in fact, the feasibility of gene replacement therapy has been shown by voretigene nepavovec, a treatment approved by the US Food and Drug Administration for inherited retinal dystrophy (IRD) caused by autosomal-recessive variants in *RPE65*.^19^ However, considering the size of its coding sequence, *ABCA4* remains a challenge for introduction via adeno-associated vectors, which are typically used for gene augmentation therapy. Since many STGD1 disease-causing variants are known to hamper the splicing process and give rise to in-frame or out-of-frame truncations or pseudo-exon insertions in *ABCA4* mRNA, antisense oligonucleotides (AONs) are a promising therapeutic strategy because of their ability to manipulate the aberrant splicing and increase the production of functional protein.

**Figure 1:**
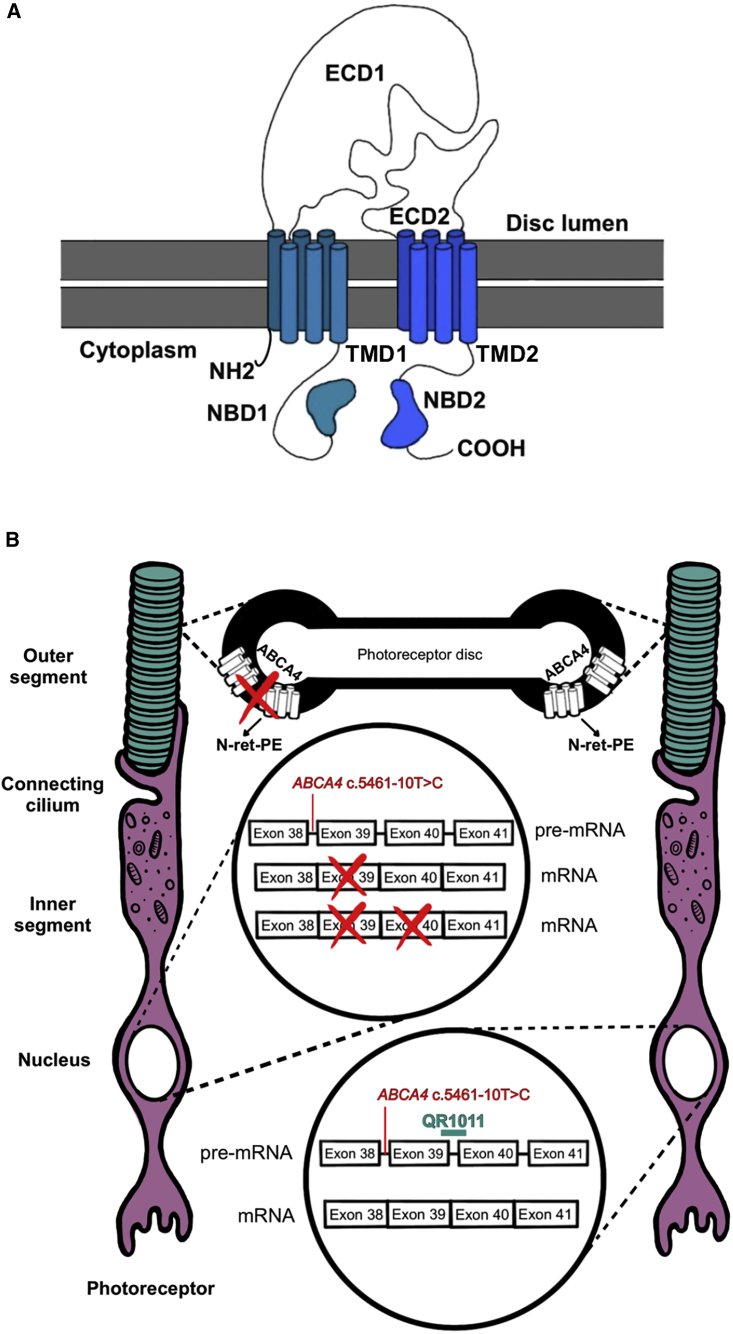
Structure of ABCA4 and the splicing modulating effect of QR-1011 (A) The complex structure of the ABCA4 protein involves two transmembrane domains (TMD1 and TMD2), each with six transmembrane helices. In addition, the protein structure displays two glycosylated extra cytoplasmatic domains (ECD1 and ECD2) and two nucleotide-binding domains (NBD1 and NBD2) where ATP hydrolysis takes place. (B) In presence of the frameshift variant *ABCA4* c.5461-10T>C, generated transcripts lack either the exon 39 or exons 39 and 40; this splicing defect hampers the production of functional ABCA4 protein and toxic retinoid products (N-retinylidene-phosphatidylethanolamine [PE]) cannot be removed from the photoreceptor’s outer segments, leading to the accumulation of A2E and lipofuscin granules, key pathogenic features for STGD1. The splicing modulating activity of QR-1011 is designed to restore wild-type splicing and include exons 39 and 40 in *ABCA4*.

AON-induced splice-modulating activity showed promising therapeutic strategies by exon exclusion, pseudo-exon exclusion, and allele-specific degradation of aberrant transcripts for several IRDs, such as autosomal recessive Leber congenital amaurosis, autosomal recessive Usher syndrome type 2A, inherited optic neuropathy, and autosomal dominant retinitis pigmentosa.^20^^,^^21^^,^^22^^,^^23^^,^^24^^,^^25^^,^^26^^,^^27^^,^^28^ The ABCA4 protein consists of two transmembrane domains that harbor six transmembrane helices each; any truncation within these sites would severely disrupt the complex protein conformation that is required for its correct function (Figure 1A).^29^ Hence, to alleviate the STGD1 phenotype, an AON-based intervention would need to redirect the reading frame to its original phase. Previous research showed AON-mediated correction of splicing defects in *ABCA4* for several deep-intronic disease-causing variants in midigene models, differentiated photoreceptor progenitor cells, and retinal organoids (ROs).^30^^,^^31^^,^^32^ To decrease the splicing defect caused by c.5461-10T>C, we designed AONs that exert their action through the mechanism of re-inclusion of skipped exons in *ABCA4* (Figure 1B). This AON-guided mechanism was successfully applied with nusinersen, the first drug approved for the treatment of spinal muscular atrophy. Nusinersen is administered intrathecally to reach the cerebrospinal fluid and it targets the intronic splice silencer to re-introduce *SMN2* exon 7.^33^ The use of this splicing manipulation for the treatment of retinal diseases has not yet been reported.

Considering the lack of animal and *in vitro* models endogenously expressing *ABCA4* c.5461-10T>C for screening therapeutic molecules, we first assessed splicing in a midigene model incorporating the *ABCA4* genomic region of interest.^34^ To assess the effect of AON treatment on ABCA4 protein expression, we employed ROs differentiated from CRISPR-Cas9-edited or patient-derived human induced pluripotent stem cells (iPSCs).^35^^,^^36^ ROs have been shown to be robust models to investigate possible therapies for various IRDs, considering that they express the targeted variants in the wider genomic environment and their retina-like lamination allows insight into the trafficking and function of disease-associated protein variants.^27^^,^^36^^,^^37^ Additionally, several transcriptomic analyses confirmed high similarities in their key characteristics with native fetal and adult human retina,^38^^,^^39^^,^^40^ which, all together, suggest the possibility of ROs replacing animal models to validate the efficacy of retina-targeted therapeutic interventions. Here, we report the ability of an AON to restore c.5461-10T>C *ABCA4* exon inclusion and ABCA4 protein in an RO model of this common variant.

## Results

### Selection of lead AON candidates in *ABCA4* minigene and midigene model

**Figure 2:**
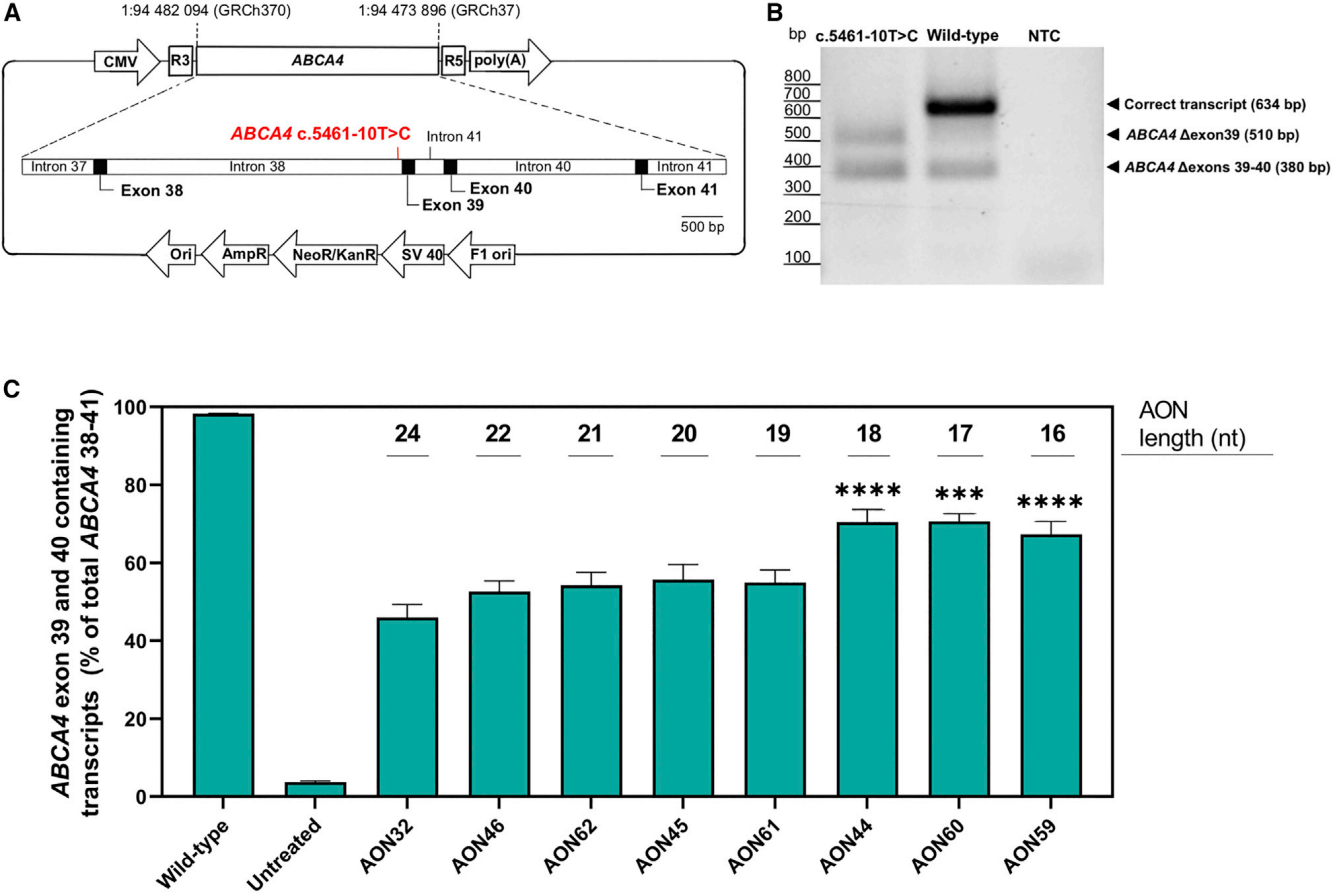
Splice-predictive midigene detected AON-induced *ABCA4* exon inclusion (A) The midigene incorporates the *ABCA4* genomic region between intron 37 and 41, together with the *ABCA4* c.5461-10T>C variant. The construct is flanked by *RHO* 3 and *RHO* 5 exons that contain strong splicing donor and acceptor sites, while the expression is initiated by the CMV promotor. (B) Expression of the *ABCA4* c.5461-10T>C midigene in HEK293 cells showed two truncated *ABCA4* isoforms: the *ABCA4* Δexon39 and *ABCA4* Δexons 39–40, but no full length *ABCA4.* The wild-type midigene displayed mostly expression of the correct transcript with a low level of the single and double skip isoforms. (C) The final AON screening with lead candidate AONs applied on midigene transfected cells demonstrated that all versions of AON32 resulted in splice correction. Data are shown as mean ± standard error of the mean, n = 6, ∗∗∗p ≤ 0.001, ∗∗∗∗p ≤ 0.0001.

We set out to identify AONs capable of correcting the splicing defect caused by *ABCA4* c.5461-10T>C. Therefore, 31 AONs were designed as an oligo-walk covering exon 39 and the surrounding intronic sequences. The AON sequences are reported in Table S1. During the sequence design, we kept optimal AON parameters when it was possible, such as a GC content of more than 40% and the Tm at greater than 48°C, as described by Aartsma-Rus,^41^ to avoid potential off-targets. *ABCA4* is retina specific, so in the absence of a cell line expressing the *ABCA4* c.5461-10T>C variant, the splice-modulating effect of the AONs was assessed in a minigene carrying the *ABCA4* exon 39 and parts of the flanking introns with c.5461-10T>C (Figure S1A), while the construct was flanked by rhodopsin (*RHO*) exon 3 and exon 5.^34^ The minigene was expressed in HEK293 cells that do not express detectable levels of *ABCA4* transcript endogenously. These cells were transfected with 100 nM AON and after 48 h; transcripts were quantified by digital droplet PCR (ddPCR). The treatment revealed an increase of exon 39 containing transcripts with AONs targeting a region at the 5′ end of intron 39 (Figure S1B) that contains strong intronic splicing silencer motifs (Figure S1C) when compared with the rest of the *ABCA4* sequence targeted by AONs (Table S2). In particular, AONs 31 and 32 managed to restore 27 ± 1.5% and 21 ± 1% of the *ABCA4* exon 39 containing transcript and served as a basis for further optimization. Since the minigene model did not contain exon 40, it cannot accurately recapitulate the splice defect observed in patient cells. In contrast, a midigene construct that incorporated a larger *ABCA4* genomic region with exons 38 and 41 (Figure 2A) showed a similar aberrant splice pattern as the one described previously in patient cells (Figure 2B).^13^ In addition, we identified low percentages of mis-spliced products lacking exon 39 and exons 39 and 40 in the wild-type midigene. This likely occurred because of the limited genomic context in the midigene and potential differences in splicing in HEK293 cells as opposed to the transcriptome data from native human retina, where this skipping was not observed (R.W.J. Collin, data not published). Further screening consisted of shorter AONs (Table S1) containing the sequence shared between AON31 and AON32 that was tested on midigene-transfected cells by transfection. Interestingly, this screening identified AON44, AON59, and AON60 as the most potent candidates that induced the greatest effect when compared with the initial molecule AON32 (Figure 2C). In addition, we noticed an increased rescue percentage when using the midigene model instead of the minigene; in fact, AON32 restored 46 ± 3% of the full-length *ABCA4* transcript in midigene-transfected cells, as opposed to the 20 ± 0.5% of rescue detected in the minigene-transfected cells. This is probably driven by the high amount of double exon skipped *ABCA4* isoform that is expressed with the midigene, but not with the minigene construct.

The dose-dependent effect of AON44, AON59, and AON60 was assessed after gymnotic administration (Figure S2). Here, the effect on splicing restoration was proportional to the concentrations of all AONs in treated samples. These experiments confirmed that shorter AONs (AON44, AON60, and AON59) could induce more splicing recovery when compared with the longer AON versions (AON32).

In addition, these four AON candidates were screened for pro-inflammatory potential *in vitro*, using a human peripheral blood mononuclear cell (PBMC) activation assay (Figure S3). The exposure of AONs at concentrations of 1 and 10 μM revealed a slight dose-dependent influence on cytokine release, which was comparable between candidates. Increases in cytokine secretion only reached statistical significance for MIP-1β, following exposure to AON32 and AON44 (10 μM), and IL-6 with interferon (IFN)-α2, after exposure to AON59 (10 μM). We observed no effect on the viability of PBMCs with any of the applied treatments (data not shown).

### Application of AON treatment on CRISPR-Cas9-edited STGD1 ROs

**Figure 3:**
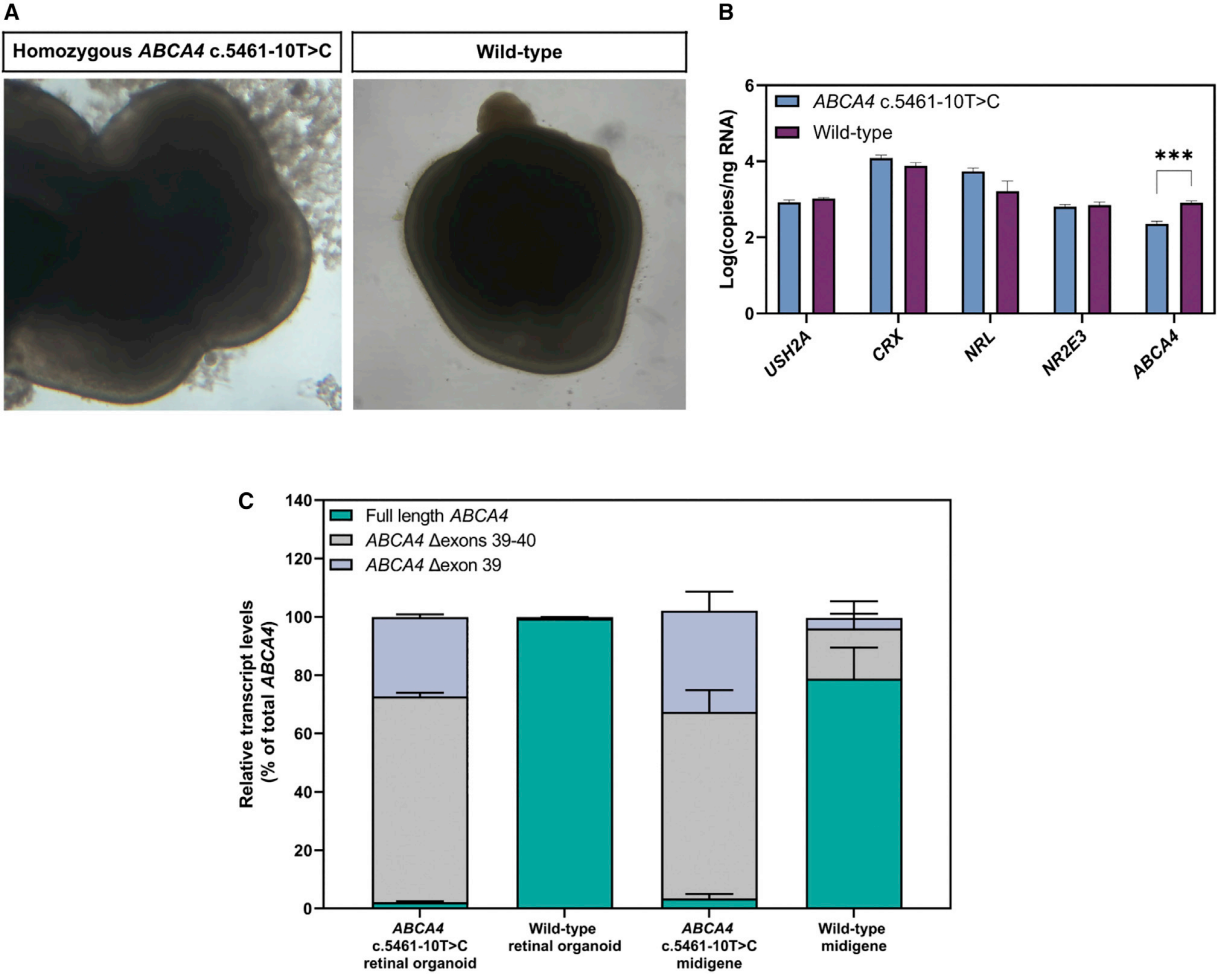
Morphological and transcript comparison of wild-type and gene-edited ROs (A) Morphology assessed at day 120 of organoid differentiation suggests ROs derived from homozygous c.5461-10T>C and the control isogenic parent cell line displayed neural retina (thin light rim at the margin of the ROs) and the brush border with photoreceptor cells. (B) Analyses of the total transcript expression of *CRX*, *NRL, NR2E3*, *USH2A*, and *ABCA4* (see Table S3). *CRX* and the photoreceptor markers were expressed similarly in both groups. *ABCA4* was expressed at higher levels in wild-type organoids. Data are shown as mean ± standard error of the mean, n = 4. (C) Comparison of *ABCA4* transcript expression in c.5461-10T>C and wild-type ROs and midigenes showed that the relative levels of the three transcripts are comparable between RO and midigene for both WT and the c.5461-10T>C variant. Data are shown as mean ± standard error of the mean, ∗∗∗p ≤ 0.0001.

Before assessing the AON treatment efficiency on ROs, we investigated the morphology and RNA content in CRISPR-Cas9-edited organoids homozygous for the c.5461-10T>C variant compared to the parent isogenic wild-type ROs. STGD1 and wild-type ROs were differentiated following the protocol published by Hallam et al.,^35^ and their morphology and transcript content were examined 120 ± 3 days after differentiation. Both groups showed a phase-light neural retina region at their edges, which is characteristic light microscopic appearance for ROs older than 90 days (Figure 3A). In addition, we observed a short brush border surrounding the margins of both groups (Figure S4A); this contains presumptive inner and outer segments of photoreceptor cells that is expected to emerge after 120 days after differentiation. Transcript analyses revealed similar expression levels of the photoreceptor precursor gene *CRX*, retinal identity marker genes (*NRL* and *NR2E3*), and *USH2A* (Figure 3B). Interestingly, the expression of *ABCA4* was significantly decreased in STGD1 organoids (Figure 3B and Table S3), suggesting the potential presence of nonsense-mediated decay that is activated by the out-of-frame RNA transcripts in STGD1 organoids. In wild-type ROs, *ABCA4* was not detected before day 35 after differentiation; the expression steeply increased until day 120 (Figure S4B), after which the increases were more gradual.

We compared the presence of the different *ABCA4* splice forms between ROs and midigenes. The c.5461-10T>C variant led to similar ratios of *ABCA4* transcripts in both organoid and midigene models: exon 38-39-40-41 isoforms were barely detected, while the Δexons39-40 isoform was the most prominent. In contrast, the wild-type ROs contained almost exclusively the correct transcript, unlike the wild-type midigene that displayed some mis-splicing (Figure 3C).

**Figure 4:**
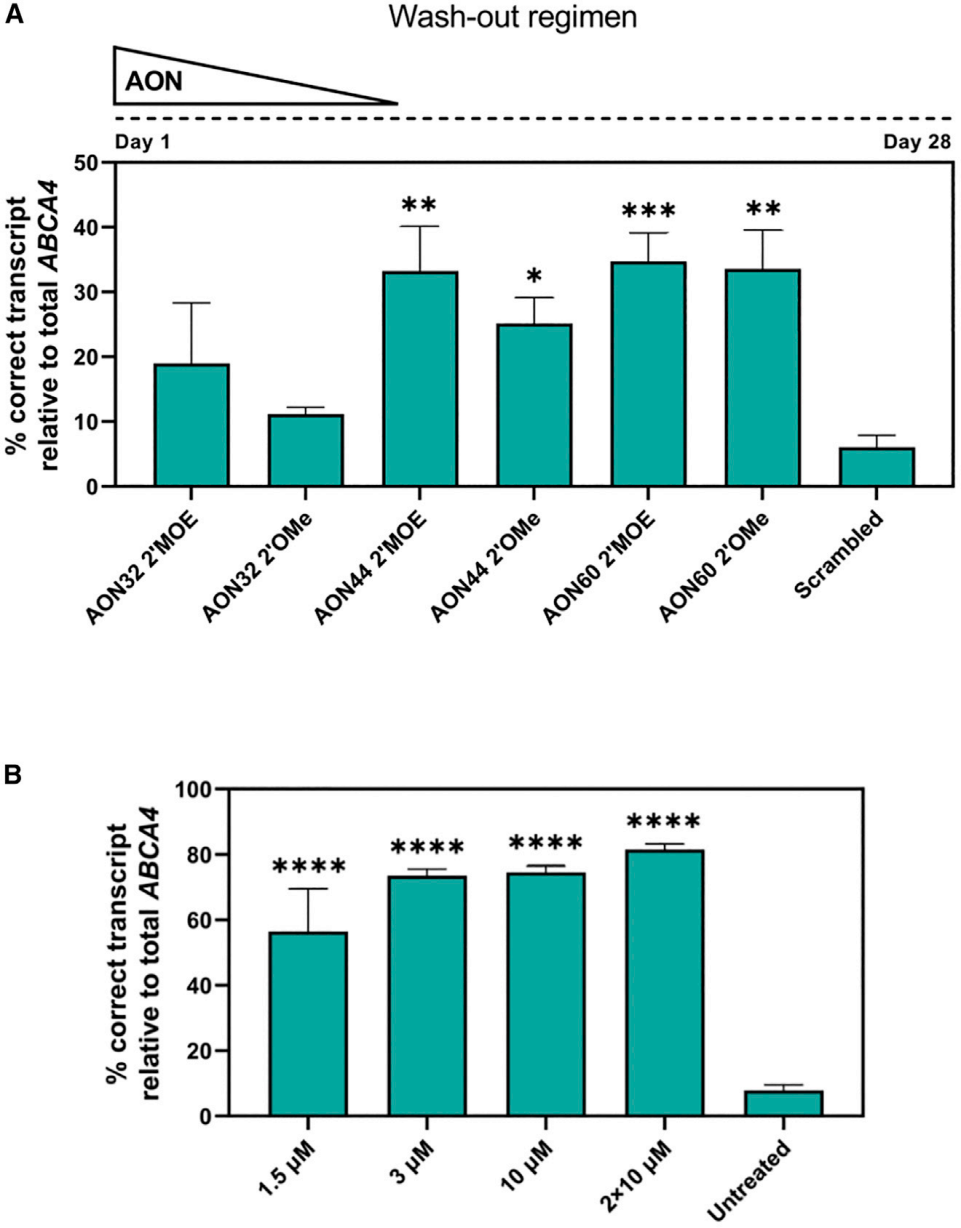
Gene-edited and patient-derived homozygous c.5461-10T>C ROs show high levels of rescued *ABCA4* transcript upon AON treatment (A) Transcript analysis of gene edited ROs treated with a 1.5 μM 2′MOE-modified and 2′OMe-modified AONs for 28 days. (Top) The 4-week-long treatment used a wash-out regimen in which the AON was added only at day 0 of treatment and its concentration was halved by each medium change every other day. (B) The percentage of splicing correction in patient-derived ROs treated with QR-1011. For the 2 × 10 μM group, QR-1011 was administered at treatment day 0 and 14. A dose-dependent increase in splice correction was observed from 21.2% to 53.4%. Data are shown as mean ± standard error of the mean, n = 6 per condition. Asterisks display the significant differences vs. scrambled oligo or untreated (∗p ≤ 0.05, ∗∗p ≤ 0.01, ∗∗∗p ≤ 0.001, ∗∗∗∗p ≤ 0.0001).

CRISPR-Cas9-edited ROs homozygous for c.5461-10T>C were treated gymnotically with AON32, AON44, and AON60 once they reached 150 days of age. The treatment followed a wash-out regimen for 4 weeks: here, the AONs were added only on day 0 of treatment at a 1.5 μM concentration, and at each 50:50 media change the AON concentration would be halved. This study included the AONs with the 2′OMe-modified sugar rings tested previously in cells; in addition, we included the same AON sequences carrying the 2′MOE sugar modifications to examine the possible difference in therapeutic effect between the two chemistries. Isoform-specific analysis revealed that AON60 and its 1-nt longer version AON44 reached 35 ± 4% and 33 ± 7% of transcript correction (Figure 4A). The detected skipping events are reported in Figure S5A. The longest molecule AON32 showed no significant improvements compared with the scrambled sample, and the two different sugar chemistries showed only small differences in outcome. Because of its superior theoretical parameters, AON44 2′MOE was selected for further analysis in ROs and named QR-1011.

### In silico analysis does not show any relevant off-target effect of QR-1011

A search against the RefSeq database showed that QR-1011 has no full complementarity to any targets in human mRNA and DNA, apart from the intended target in *ABCA4*. We also identified no target with one mismatch, whereas one coding region in *MGRN1* showed to be a potential off-target with two mismatches (GRCh37, chr16:4683901-4683916). In addition, potential off-targets in genomic DNA with 2 mismatches were predicted for 5 intergenic and 16 deep-intronic regions (Table S4); since the distance of the flanking exons for all genes was 225 bases or more, it was concluded that the possible interference with the splicing of these genes by QR-1011 was unlikely.^42^ Given the short size of QR-1011, a near perfect match would be necessary for efficient hybridization and it is, therefore, highly unlikely that the oligo would efficiently hybridize to targets with two or more mismatches, as reported previously.^43^

### Range of activity of lead candidate QR-1011 in patient-derived ROs homozygous for *ABCA4* c.5461-10T>C

Based on previous studies (K.D., unpublished data), it was noticed that longer treatment periods up to 3 months enable more AON-mediated transcript correction, potentially related to endosomal storage and release of AON, which could consequently lead to the production of more wild-type protein. Hence, the next study consisted of a wash-out treatment 8 weeks long where QR-1011 was applied at four different concentrations. Homozygous c.5461-10T>C patient-derived iPSCs^7^ were differentiated to ROs, QR-1011 was administered at D180 at concentrations of 1.5 μM, 3 μM, and 10 μM, and the organoids were harvested 56 days later. To explore the relative efficacy of QR-1011 dosing and wash-out, some of organoids treated with the 10-μM dose were retreated with another 10-μM dose 14 days after the first dose and the treatment continued for 6 additional weeks. The isoform analysis revealed that the 1.5-μM dose of QR1011 reached 56 ± 13% correct exon 38-39-40-41 *ABCA4* transcript of the total detected *ABCA4* (Figure 4B). Interestingly, the organoids that underwent the 10-μM and the 2 × 10-μM treatments did not show significantly higher restoration of splicing when compared with the group treated with 3 μM QR-1011. Indeed, a post-treatment analysis of RNA indicated that the AON activity at 3 μM restored 74 ± 2% of correct splicing and reached a plateau phase, suggesting this dose potentially corrected most of the available aberrant transcript. This experiment also showed that an 8-week-long treatment induced more AON-mediated correction when compared with a 4-week-long treatment; moreover, the 1.5-μM dose here induced 41% more correctly spliced *ABCA4* than the previous experiment where the same concentration of AON was administered (Figure 4A). In addition, we noticed that the untreated patient-derived ROs displayed slightly higher, yet not significantly different, amounts of correct transcript (8 ± 2%), compared with those detected previously in the CRISPR-Cas9-modified organoids (6 ± 2%). The percentage of measured skipping events are shown in Figure S5B.

### Patient-derived ROs display a dose-response splicing rescue of RNA that correlates with wild-type ABCA4 protein rescue

**Figure 5:**
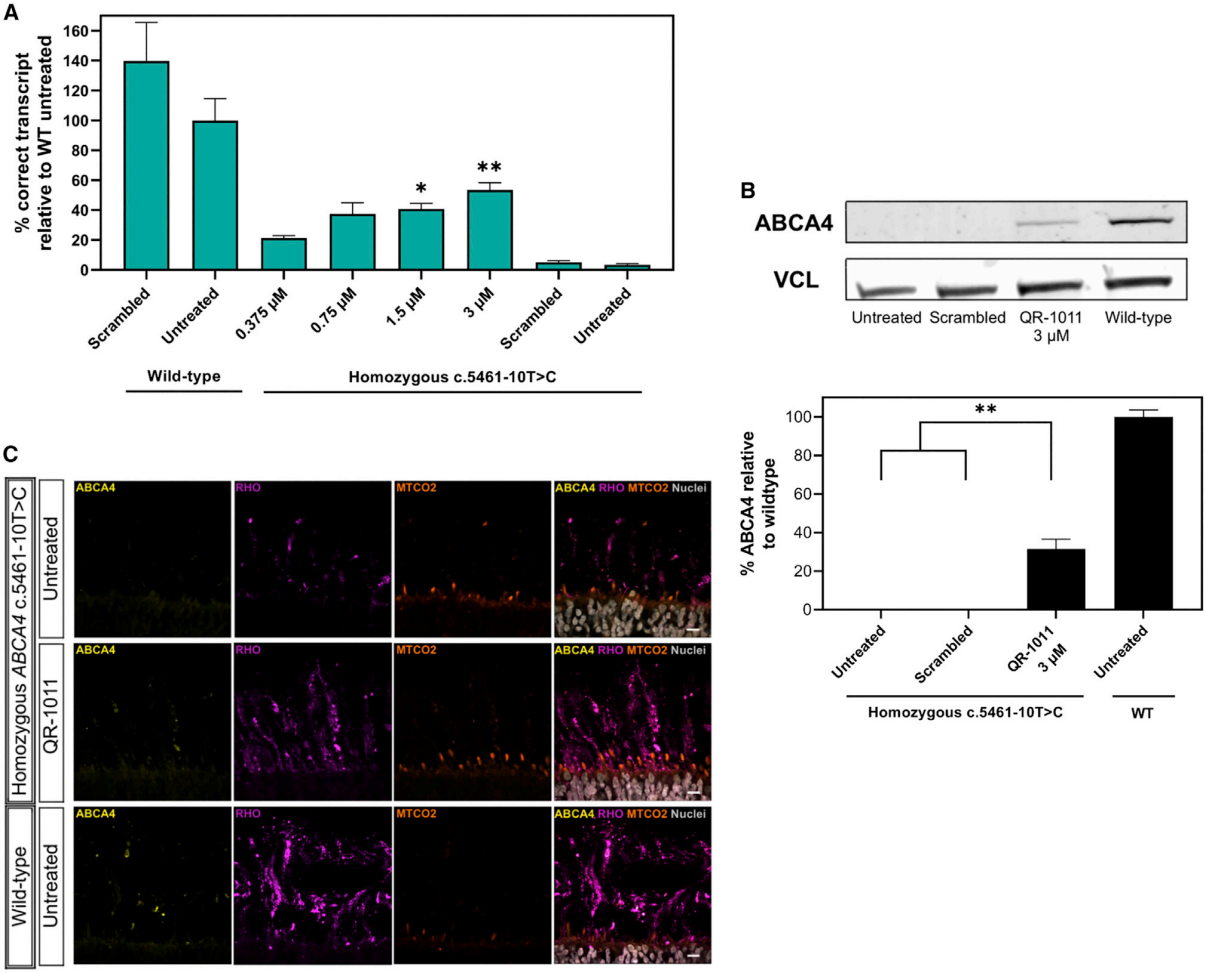
Low concentrations of QR-1011 can restore RNA splicing and rescue of the wild-type protein in patient-derived c.5461-10T>C ROs (A) Splice adjustment efficacy with clinically relevant dosages of QR-1011 in ROs deriving from a biallelic c.5461-10T>C patient cell line. After 56 days of treatment, all patient-derived RO samples showed splice restoring activity of QR-1011, while the scrambled AON showed no effect on *ABCA4* splicing. Data are shown as mean ± standard error of the mean, n = 6. (B) Western blot analysis (n = 3) of treated patient-derived ROs and wild-type ROs revealed restoration of ABCA4 protein after QR-1011 treatment. Untreated c.5461-10T>C ROs and those subjected to treatment with scrambled AON contained no detectable protein. All samples were normalized to the average signal obtained from wild-type ROs. Vinculin (VCL) was used as a loading control. Data are shown as mean ± standard error of the mean, n = 3. ∗p ≤ 0.05, ∗∗p ≤ 0.01. (C) ABCA4 protein immunoreactivity (yellow) in treated patient-derived organoids colocalized within the outer segments (OS, rhodopsin, magenta) of photoreceptor cells and resembled the localization found in wild-type ROs. The inner segments (IS) were visualized with the mitochondrial-targeting antibody MTCO2 (orange) and DAPI nuclear staining is shown in gray. Scale, 10 μm.

To assess the activity of QR-1011 at lower concentrations, patient-derived ROs were treated with QR-1011 at concentrations of 0.375 μM, 0.75 μM, 1.5 μM, and 3 μM. The treatment followed the same design applied in the previous experiment described above. This treatment included two positive control groups of wild-type ROs that were either treated with a scrambled AON or left untreated. The aim of this experiment was to compare the restoration of *ABCA4* levels with those observed in wild-type ROs, in contrast with previous treatments, where the AON effect was calculated as part of total *ABCA4* within the same sample. Eight weeks after treatment, the *ABCA4* 38-39-40-41 transcript content showed a clear dose response in patient-derived ROs; an increase to 21 ± 1.5% in the generation of correct *ABCA4* isoform was detected with the lowest 0.375 μM dose of QR-1011, whereas the highest 3 μM dosage rescued 53 ± 5% of the correct transcript when compared with the total *ABCA4* content detected in untreated wild-type ROs (Figure 5A). The remaining *ABCA4* isoforms detected in these ROs are reported in Figure S5C.

To assess whether the presence of an off-target oligo interferes with the transcript content, we investigated whether the wild-type untreated ROs and those that underwent treatment with the scrambled AON showed a significant difference in their total expression of *ABCA4*. Predictably, statistical analysis of these two groups did not indicate any significant difference in the expression or splicing of *ABCA4*.

Western blot analyses were performed on a pool of patient-derived ROs treated with 3 μM QR-1011 and compared with ROs that were untreated or treated with scrambled AON (used as negative controls) and untreated wild-type ROs that served as positive controls. The antibody directed against the N-terminus of the protein revealed AON-induced rescue of the wild-type protein in biallelic variant ROs; these expressed 32 ± 5% of newly generated protein relative to the wild-type organoids (Figure 5B). Additional details regarding the western blot analysis can be found in Figure S6 and Table S5. ABCA4 protein was undetectable in untreated homozygous c.5461-10T>C ROs, correlating with the very low levels of the correct in-frame transcript.

To investigate the trafficking and the subcellular expression of the newly generated ABCA4 protein in more detail following AON treatment, cryosections of ROs were investigated by immunohistochemistry. The fragile outer segments of photoreceptor cells surrounding the ROs were preserved with gelatin embedding, as described previously.^44^ Wild-type ABCA4 was visualized by immunofluorescence with an ABCA4 antibody targeting the C-terminal part of the protein. We observed that, in wild-type ROs, ABCA4 immunoreactivity was exclusively in the outer segments of the photoreceptor cells co-stained with rhodopsin antibody. Interestingly, 3 μM-treated patient-derived ROs displayed ABCA4 immunoreactivity in the photoreceptor outer segments, as opposed to the untreated patient ROs, where no immunoreactivity was detected (Figure 5C). This staining confirmed that the trafficking of the rescued ABCA4 protein is in line with what is observed in the native mammalian retina.^45^ We did not detect any protein retention in the inner segment of photoreceptor cells that was stained with a mitochondria-targeted antibody against MTCO2. The same localization of ABCA4 immunoreactivity was observed in wild-type ROs. These findings suggest that the AON-mediated treatment not only restores wild-type ABCA4 protein, but the protein generated upon AON treatment is also trafficked to the expected subcellular compartment.

## Discussion

In this study, we report the development and validation of target-specific AONs as a possible therapeutic approach to correct the aberrant splicing of *ABCA4* caused by the severe STGD1-causing variant c.5461-10T>C. This variant was previously identified as a non-canonical splice site variant that induces the generation of deleterious transcripts (lacking either the single exon 39 or exons 39 and 40) that lead to frameshifts in the open reading frame. Extended *in vitro* AON screenings in a c.5461-10T>C midigene model identified potent lead candidates that were able to correct 70% of the aberrant splicing. These molecules were validated in differentiated ROs where we observed high levels of splicing correction accompanied by rescued wild-type ABCA4 protein.

AONs have demonstrated promising results in several pre-clinical studies for IRDs by correcting pathological RNA processing events associated with entire exon skipping, complete degradation of abnormal transcripts, or pseudo-exon exclusion. In the clinic, AON-mediated splicing therapy was well tolerated and able to significantly improve the best-corrected visual acuity in phase I clinical trials with sepofarsen (LCA10) and ultevursen (*USH2A*-associated retinitis pigmentosa and Usher syndrome).^28^^,^^46^^,^^47^ These AONs delay disease progression through pseudo-exon and in-frame exon skipping, respectively. The efficacy of both compounds is currently being investigated in phase II and III clinical trials.

Considering that the ABCA4 protein is a complex membrane protein constituted of 12 transmembrane helixes that are involved in the transport of substrates,^29^ many of the disease-causing variants are predicted or known to lead to protein misfolding.^48^ Splicing variants in *ABCA4* are estimated to comprise 25% of all STGD1-causing variants, which emphasizes the importance of the advancement of AON-mediated therapy because of its splicing-manipulating activity. Here, AON-mediated exon inclusion was implemented to re-include skipped exons 39 and 40 in *ABCA4* and correct the disrupted splicing caused by *ABCA4* c.5461-10T>C. This AON mechanism of action could be of major importance in the development of therapies for STGD1, since the complexity of the ABCA4 protein structure suggests that it is unlikely that major truncation of the original amino acid sequence would be tolerated without leading to a disease phenotype.^30^ The potential of AON-based re-inclusion of skipped exons as therapy has been illustrated by several pre-clinical studies for Pompe disease, cystic fibrosis, and Alzheimer’s disease.^49^^,^^50^^,^^51^ All reported a significant post-treatment functional rescue that is a prerequisite for ameliorating disease-associated phenotypes. The most effective AONs from these studies block intronic splice silencers (ISSs) located in the adjacent introns to promote the exon inclusion. Similarly, QR-1011 is designed to block three strong ISSs located in intron 39 of *ABCA4* and restore the canonical splicing (Figure S1C).

*ABCA4* c.5461-10T>C is considered the most common severe variant that underlies STGD1.^8^ Even though the clinical features associated with STGD1 reveal a wide range of heterogeneity, the severity of *ABCA4* variants is directly correlated with the onset of the disease.^52^ The −10T>C variant is more often found in combination with one other moderate or mild variant in *ABCA4* than in a homozygous state.^6^^,^^8^ Considering that severe mutations lead to the early STGD1 onset, where the progress of the disease is faster, and milder mutations are associated with slower development of STGD1 hallmarks,^53^^,^^54^ the advanced stage of STGD1 could be significantly postponed or even completely repressed by correcting the aberrant splicing due to c.5461-10T>C. In the case of two deleterious *ABCA4* alleles, the disease-associated changes characteristic of the early stage are observed in the region limited to the macula. The most progressed phases of the disease exhibit severe degenerative lesions of the retina that extend across the posterior pole of the retinal tissue and cause severe visual impairment. The loss of photoreceptor cells in STGD1 prevents the reversion of the disease-associated phenotype; however, individuals diagnosed in an early stage of the disease could benefit greatly from the AON-based intervention, since this would slow or stop the progress of advanced STGD1 features. Proof of concept acquired from patient-derived ROs show that QR-1011 was effective in restoring correct *ABCA4* transcript splicing, followed by the production of the wild-type protein. Regarding its safety profile, high concentrations of QR-1011 did not exhibit overt toxicity throughout the screenings in cells and studies in ROs. The modest influence on the secretion of pro-inflammatory cytokines and chemokines upon exposure to PBMCs suggests a favorable immunostimulatory profile. Nevertheless, further dedicated toxicology studies to completely assess the potential adverse effects of the molecule will be required. In contrast, low QR-1011 concentrations, ranging from 0.375 to 3 μM, were able to correct the transcript and higher amounts of aberrant splicing correction were observed in longer organoid treatments, compared to shorter treatments. We noticed that 4-week-long treatments were less effective than 8-week-long treatments, even with the same AON concentrations. This suggests that the therapeutic effect does not stop soon after exposure to QR-1011, but is rather more durable and might prolong the interval between the doses once in clinic. The likely mechanism behind this event involves the slow endosomal release of the AON to the nucleus after its entrapment in early or late endosomes upon endocytosis.^55^ Future studies on non-human primates will be required to further assess the tolerability and the safety profile of QR-1011, which can determine the effective clinical dose for intravitreal delivery. These should be accompanied by investigations of the pharmacokinetic properties of the molecule to establish the optimal dosing interval.

The characterization of disease-associated clinical features and investigation in novel therapies demand robust and credible pre-clinical models. ROs differentiated from pluripotent stem cells have demonstrated promising *in vitro* applications; several groups reported protocols for the generation of three-dimensional (3D) layered retina-like tissues.^56^ To evaluate the splice-regulating effect of our AONs, we used CRISPR-Cas9-edited and patient-derived ROs biallelic for *ABCA4* c.5461-10T>C. These STGD1 organoids were differentiated using two different protocols published by Hallam et al.^35^ and Hau et al.^57^ Once they reached the mature stage 3 after 120 days of differentiation, the ROs seemed to be similar, despite differences in differentiation procedures. They had similarities in lamination accompanied by a developed surrounding brush border. In addition, both protocols produced ROs that morphologically resembled the wild-type ROs, despite the presence of the severe *ABCA4* variant on both alleles. The RNA content of most retinal markers was concordant between all types of ROs, with the exception of *RHO*, which was detected at significantly higher levels in patient-derived and controls ROs produced with the adherent non-embryoid body method by Hau et al.^57^ than in gene-edited and control ROs produced with the Hallam et al.^35^ method. In addition, we observed a reduced level of *ABCA4* transcript in STGD1 organoids, as opposed to wild-type ROs, which is most likely due to nonsense-mediated decay of the exon-skipping transcripts in STGD1 organoids. To study the expression and localization of the wild-type protein in detail, we conducted protein analysis by western blot and immunohistochemistry. Considering the fragile nature of the photoreceptor outer segments, we used embedding in gelatin, which previous studies reported improved the preservation of structures surrounding the ROs.^44^ The ABCA4 protein was clearly expressed in wild-type ROs and its localization was confined to the outer segments of retinal photoreceptors, as observed in the human retina. ABCA4 was much less present in the outer segment when compared with rhodopsin, which is in line with earlier observations conducted in mouse rod cells where the molar ratio of ABCA4 to rhodopsin was 1:300.^58^ The protein assays in STGD1 ROs that underwent the AON treatment confirmed the usefulness of ROs as *in vitro* model for STGD1; we confirmed that the rescued protein is trafficked and co-localizes in outer segments of photoreceptor cells. As reported previously, the correct subcellular localization of rescued protein is important for alleviation of the STGD1 phenotype.^59^ The amount of ABCA4 residual activity required to prevent the STGD1 phenotype in affected individuals is not precisely determined. Theoretical modeling studies (F.P.M. Cremers, personal communication, 2023) suggest that the threshold of remaining ABCA4 activity in an individual, below which STGD1 will develop, is estimated at between 30% and 40%. Previous inquiries on *abca4*^−/−^ mice identified significantly decreased traces of deposited lipofuscin after compensating just 10% of wild-type protein.^60^ In addition, the activity of isolated and purified protein derived from wild-type and several disease-causing missense variants has been determined^61^^,^^62^; these studies suggest the basal activity of severe variants to be less than 25% of the wild-type protein.^48^ Importantly, we quantified the restored ABCA4 protein after treatment with QR-1011 at 32 ± 5% of the wild-type levels, as opposed to untreated biallelic c.5461-10T>C samples, where no protein was detected.

In conclusion, QR-1011 showed robust therapeutic potential by correcting high levels of truncated transcripts in *ABCA4* c.5461-10T>C when administered to both midigene-transfected cells and 3D human ROs. In 8-week-long treatment periods, the AON-corrected transcripts led to the production of the wild-type ABCA4 protein, which trafficked to the outer segments of photoreceptor cells in ROs. The measured amounts of rescued RNA and protein suggest the AON effect would be sufficient to alleviate the STGD1 phenotype, and therefore QR-1011 shows potential as a therapeutic strategy for the most common severe STGD1-causing variant *ABCA4* c.5461-10T>C.

## Materials and methods

### Generation of *ABCA4* wild-type and *ABCA4* c.5461-10T>C minigenes and midigenes

pIC-neo.Rho3-5.MCS^63^ was generated by replacing the *USH2A* sequences of pCI-neo.Rho.USH2A-PE40-wt by a custom MCS containing the following restriction enzyme recognition sites: XhoI, EcoRI, MluI, EcoRV, XbaI, SalI, and Cfr9I designed to aid in downstream cloning steps. The custom MCS was generated by annealing DNA oligonucleotides 5′-CTCGAGAATTCACGCGTGGTGATATCACCTCTAGAGTCGAC-3′ and 5′-CCCGGGTCGACTCTAGAGGTGATATCACCACGCGTGAATTCT-3′. The resulting fragment was used in a ligation mixture together with the backbone plasmid, digested with XhoI and Cfr9I (Thermo Fisher Scientific, Waltham, MA, USA).

To generate a *ABCA4* c.5461-10T>C minigene, the pCI vector backbone and a synthetic dsDNA sequence (gBlock; Integrated DNA Technologies, Coralville, IA, USA) containing the *ABCA4* minigene genomic region with exon 39 and parts of adjacent introns (1:94,477 466–94,476 525, GRCh37) (Figure S1A) and the c.5461-10T>C mutation were digested using the *Eco*RI (New England Biolabs, Ipswich, MA, USA) and *Sal*I (Thermo Fisher Scientific). The digested vector was loaded on 1% agarose gel, isolated and purified using the Nucleospin Gel and PCR Clean-up kit according to the manufacturer’s instructions (MACHEREY-NAGEL, Düren, Germany). The digested gBlock was purified directly using the same kit. Digested fragments were ligated overnight at 16°C with T4 ligase (Thermo Fisher Scientific) following the manufacturer’s protocol. The ligation reaction was used to transform DH5α-competent cells (Thermo Fisher Scientific) according to manufacturer’s protocol.

To generate an *ABCA4* wild-type midigene (construct with >1 exons and surrounding introns), genomic DNA from HeLa cells (ATCC, Manassas, VA, USA) was extracted with the DNeasy Blood & Tissue Kit (QIAGEN, Hilden, Germany). The *ABCA4* genomic region between intron 37 and intron 41 (1:94,482 094–94,473 896, GRCh37) (Figure 2A) was amplified with primers (Integrated DNA Technologies) containing recognition sites for *Eco*RI and *Sal*I, using the Phusion High-Fidelity DNA Polymerase kit (Thermo Fisher Scientific) according to the manufacturer’s protocol. The wild-type PCR product and the digested pCI vector backbone were ligated and transformed into GT115 competent cells (InvivoGen, San Diego, CA, USA). To introduce the *ABCA4* c.5461-10T>C mutation, the wild-type plasmid and a gBlock (Integrated DNA Technologies) from intron 37 to intron 41 containing the c.5461-10T>C mutation were digested with *Box*I and *Bsi*WI (Thermo Fisher Scientific) and subsequently ligated and transformed into GT115-competent cells.

### AON screening in HEK293 cells

HEK293 cells (ATCC) were cultured with DMEM (Life Technologies, Waltham, MA, USA) with 10% Fetal Bovine Serum (Biowest, Nuaillé, France) at 37°C with 5% CO_2_. We transfected 2 × 10^5^ cells with 50 ng of either *ABCA4* minigenes or midigenes with Lipofectamine 3000 Transfection Reagent (Thermo Fisher Scientific) by following the manufacturer’s protocol. The AON was delivered by transfection or gymnotically as described by Dulla et al.^47^ The RNA was extracted 48h post-treatment with RNeasy Plus Mini Kit (QIAGEN) and 500 ng was reverse transcribed using the Verso cDNA Synthesis Kit (Thermo Fisher Scientific) by following the manufacturer’s protocol. We analyzed 5 ng cDNA with isoform-specific droplet digital PCR assays and ddPCR Supermix for probes (Bio-Rad, Hercules, CA, USA). Primers and probes (Integrated DNA Technologies) used for dPCR are listed in Table S6. The following PCR program was used: enzyme activation at 95°C for 10 min (1 cycle), denaturation at 95°C for 30 s and annealing/extension at 60°C for 1 min (40 cycles), and enzyme deactivation at 98°C for 10 min (1 cycle). The fluorescence signal of individual droplets was measured in the QX200 Droplet Reader (Bio-Rad). In each experiment, the thresholds to separate the positive droplet population from the negative were set manually. The following formulas were used to calculate the percentage of *ABCA4* exons 39–40 inclusion isoform in minigene: Total *ABCA4* = *ABCA4* exon39 inclusion + *ABCA4* Δexon39; Correct transcript % relative to total *ABCA4 = ABCA4* exon39 inclusion/Total *ABCA4*.

The formulas that were used in the midigene model and ROs were the following: Total *ABCA4* = (*ABCA4* exons 39–40 inclusion) + (*ABCA4* Δexon39) + (*ABCA4* Δexons39–40); Correct transcript % relative to total *ABCA4* = (*ABCA4* exons 39–40 inclusion)/Total *ABCA4*.

### In silico analysis of possible off-target effect of selected lead AON molecules

*Homo sapiens* RefSeq RNA was downloaded from the NCBI website and used for finding potential off-targets of QR-1011 (ftp.ncbi.nlm.nih.gov/refseq/H_sapiens/mRNA_Prot/). *H. sapiens* gene sequence database was generated from human genome sequence database (ftp.ensembl.org/pub/release-90/fasta/homo_sapiens/dna/) using Ensemble gene annotations (ftp.ensembl.org/pub/release-90/gff3/homo_sapiens/). *H. sapiens* mRNA and pre-mRNA sequence databases were queried using a custom made bioperl program. The reverse complement sequence of QR-1011 (CCGAGGCCCATGGAGCAT) was used for the searches. In cases where QR-1011 matched with multiple mRNA isoforms of the same gene, it is considered as one match/target and match with highest complementarity (least mismatches) is reported. The Ensemble Genome Browser (http://www.ensembl.org/index.html) was used to find the exact location of the match and to calculate the distances to the flanking exons. The target gene was queried against human sequences. From the results, the most abundant transcript was selected and the target sequence was searched.

### Assessment of the immunostimulatory and cytotoxic potential of lead AON candidates AONs in PBMCs

Buffy coats, the fraction of an anti-coagulated blood sample that contains most of the white blood cells and platelets after the centrifugation of the blood (500 mL blood in 70 mL citrate phosphate dextrose coagulant), from five healthy human (consensual) blood donors, were obtained from Sanquin Blood Supply in Rotterdam, the Netherlands. PBMCs were isolated from each buffy coat within 24 h after blood collection, aliquoted, and cryopreserved. PBMCs were stimulated for 48 h with QR-1011 candidates AON32, AON44, AON59, or AON60 at a concentration of 1 μM and 10 μM; positive control R848 (1 μM); or PBS (vehicle control) at 37°C under a 5% CO_2_ atmosphere. For every donor, all conditions were tested in triplicate in 96-well round-bottom microtiter plates. The total number of viable PBMCs per well was 3 × 10^5^. R848 (Resiquimod [tlrl-r848]; InvivoGen, San Diego, CA, USA), a potent Toll-like receptor (TLR)7/8 agonist, was selected as a positive control for its strong and robust immune-activating properties, inducing the production of pro-inflammatory cytokines. Also, R848 acts on the TLRs that are most likely to be involved in recognition of single-strand RNA, arguably making it the most relevant positive control for this purpose. After incubation, cell culture supernatant was isolated following centrifugation (300 relative centrifugal force for 5 min at room temperature). The viability of PBMCs after exposure to test items was assessed by resazurin reduction assay (CellTiter-Blue Reagent; Promega, Madison, WI, USA). Cytotoxicity was assessed by measurement of lactate dehydrogenase in the cell culture supernatant (CyQUANT LDH Cytotoxicity Assay; Thermo Fisher Scientific). Readout of viability and cytotoxicity assays was performed on a SpectraMax M5 Microplate reader. Cytokine levels in PBMC culture supernatants were measured using the MILLIPLEX MAP Human Cytokine/Chemokine Magnetic Bead Panel-Custom 6 Plex-Immunology Multiplex Assay (Merck KGaA, Darmstadt, Germany). Analytes included IFN-α2, IL-6, IP-10, MIP-1α, MIP-1β, and tumor necrosis factor-α. Assay plates were read on the Luminex MAGPIX platform (Luminex, San Francisco, CA, USA). Analysis of the Luminex data was performed in Bio-Plex Manager 6.1 software (Bio-Rad). Standard curves were fitted using five-parameter logistic regression. Cytokine concentrations that were outside of the detectable range of the assay were imputed for the purpose of calculation and statistical analysis. Values below the limit of detection (LOD), rendered “out of range <” by the analysis software, were imputed with a concentration value of ½ × LOD. The LOD values, which were empirically determined by the manufacturer of the Luminex kit, were derived from the technical data sheet. Conversely, cytokine concentrations that were above the upper limit of quantification, rendered “out of range >” by the analysis software, were imputed with a concentration value of two times the concentration of the highest calibrator.

### Generation of wild-type and homozygous *ABCA4* c.5461-10T>C ROs

GibcoTM Episomal hiPSCs line #A18945 was CRISPR-Cas9-edited by the manufacturer (Thermo Fisher Scientific) to carry the *ABCA4* c.5461-10 T>C variant on both alleles. The wild-type and mutant iPSCs were cultured on Matrigel hESC-Qualified Matrix coating (Corning, Corning, NY, USA) with mTeSR1 medium (STEMCELL Technologies, Vancouver, Canada) and 1 × mTeSR1 Supplement (STEMCELL Technologies). Patient-derived homozygous c.5461-10T>C iPSCs were generated previously upon approval by the institutional review board, following the tenets of the Declaration of Helsinki.^7^ The iPSCs were differentiated in ROs following the protocol described by Hallam et al.^35^ or Hau et al.^57^

### AON treatment in ROs

ROs were treated with AONs after at least 150 days of differentiation. At treatment initiation, the culture medium was fully removed and fresh medium containing AON was added. Every 2 days, one-half of the culture medium was replaced by fresh culture medium, resulting in a gradual decrease in AON concentration in the medium. Eight weeks after treatment, the culture medium was removed, the ROs were washed in PBS. and 300 μL of TRIreagent (Zymo Research, Irvine, CA, USA) was added. The samples were snap-frozen in liquid nitrogen and stored at −80°C until RNA extraction.

After thawing, the organoids were lysed by passing through a 25G needle (Henke Sass Wolf, Tuttlingen, Germany) until homogenized, and the RNA was extracted with the Direct-Zol RNA MicroPrep kit (Zymo Research); 80 or 100 ng RNA were reverse transcribed as described above, and 5 ng cDNA was analyzed with isoform-specific dPCR assays and QIAcuity Probe PCR Kit according to manufacturer’s instructions (QIAGEN) in either 26k 24-well or 8.5k 96-well Nanoplates (QIAGEN). The plates were analyzed in a QIAcuity digital PCR instrument, using the following PCR program: enzyme activation at 95°C for 2 min (1 cycle), denaturation at 95°C for 15 s, and annealing/extension at 60°C for 30 s (40 cycles). The number of different isoforms was quantified by image acquisition of wells according to the selected detection channels in the experiment setup.

### RNA analysis in ROs

Retinal markers *CRX*, *OPN1MW*, and *RHO* were used for quality control of ROs; the thresholds were set at more than 1,000 copies/ng RNA. The samples that were below two of the three thresholds were excluded from the analysis. The thresholds for separation of positive and negative partitions were manually set in all experiments. To correct for different cDNA input, the three identified *ABCA4* isoforms were normalized to the geometric mean of *CRX, RHO*, and *OPN1MW* and the percentage of correct *ABCA4* transcript was calculated as relative to total *ABCA4* within each sample or as relative to total *ABCA4* in wild-type untreated organoids when these were included in the experiment: Correct transcript % relative to total *ABCA4* = (*ABCA4* exons 39–40 inclusion)/Total *ABCA4*; Correct transcript % relative to total *ABCA4* in wild-type untreated = (*ABCA4* exons 39–40 inclusion)/Total *ABCA4* (wild-type untreated) × 100.

### Immunohistochemistry

The AON-treated ROs were fixed in 2% paraformaldehyde (Thermo Fisher Scientific) and 5% sucrose (Thermo Fisher Scientific) for 15 min at 4°C, followed by a 30-min incubation in 7.5% sucrose, 30 min in 15% sucrose, and 2 h incubation in 30% sucrose. The organoids were transferred to a cryomold and embedded in 7.5% gelatin (porcine skin; Merck KGaA) and 10% sucrose. The sample blocks were then frozen at −80°C. Sections of 10 μm thickness were sliced on a Cryotome FSE (Thermo Fisher Scientific), rehydrated in PBS, and stained following the protocol described by Cowan et al.^46^ ABCA4 was detected using the anti-ABCA4 3F4 clone (1:100; Abcam, Cambridge, UK), rhodopsin was stained using the anti-rhodopsin 4D2 clone (1:300, Invitrogen), mitochondria were detected with an anti-MTCO2 antibody (1:150; Abcam), and nuclei were stained with Hoechst 33,342 (1:1000; Thermo Fisher Scientific). Images were collected on an LSM 800 confocal microscope (Carl Zeiss, Oberkochen, Germany) using a 60× objective and analyzed with ZEN Blue edition (Carl Zeiss) using the maximum intensity projection.

### Identification of protein rescue by western blotting

The ROs were pooled (10) and lysed in radioimmunoprecipitation assay protein lysis buffer (Abcam) with protease inhibitor cocktail (Roche, Basel, Switzerland) and homogenized using a 25G needle. The protein concentration was assessed using the Pierce BCA Protein Assay Kit (Thermo Fisher Scientific) according to the manufacturer’s instructions and the plate absorbance was read in the SpectraMAX plate reader (Molecular Devices, San Jose, CA, USA) at 562 nm. The samples (22.5–85 μg) were loaded on 4–20% Mini-PROTEAN TGX Precast Protein Gels (Bio-Rad), ran for the first 30 min at 70 V and the next 4 h at 100 V in Tris-Glycine SDS buffer. The gels were transferred to PVDF membranes (Merck KGaA) previously activated with methanol, in 1× Tris-Glycine buffer and 20% methanol at 70 mV overnight at 4°C. The membranes were rinsed in PBS-0.1% Tween, blocked in Pure Odyssey Blocking Buffer (Li-COR Biosciences, Lincoln, NE, USA) for 2 h and incubated with an anti-ABCA4 clone 5B4 (1:1,000; Merck KGaA) and anti-vinculin (1:5,000; Abcam) at 4°C overnight. The membranes were washed with PBS-0.1% Tween and incubated with Goat Anti-Mouse IRDye 800 and Goat Anti-Rabbit IRDye 680 (1:5000; Li-COR Biosciences) for 1.5 h in the dark. The membranes were washed with PBS-0.1% Tween and scanned wet in the Odyssey IR system (Li-COR Biosciences). The intensity of the detected bands was quantified using FIJI ImageJ 1.53c, and the samples were normalized to the wild-type sample. The mean percentage of the detected ABCA4 protein was statistically analyzed using GraphPad Prism 9, with ordinary one-way ANOVA test followed by Dunnet’s multiple comparison test.

### Statistical analysis

All graphs represent data as mean ± standard error of the mean. Significant differences in gene expression reported in Figure 3B and Table S3 were identified using multiple unpaired t test. For comparisons between multiple groups, ordinary one-way ANOVA was used, followed by Tukey’s multiple comparison test (Figure 2C) or Dunnet’s multiple comparison test (all other Figures).

Before statistical comparison of cytokine secretion data, outlier removal was performed for every treatment condition, except for positive control R848 using the “Identify Outlier” option, using ROUT method with a Q-value of 0.5%. Subsequently, log-transformed cytokine concentration values were subjected to matched comparison to PBS-treated controls using mixed-effects analysis, correcting for multiplicity using Dunnett’s correction.

The statistical analyses were performed with GraphPad Prism 9, and a p value of 0.05 or less was considered statistically significant.

## Data availability

All data generated throughout this study are available within the paper and its supplemental information. Raw data are available upon request from corresponding authors.

## Supplemental information

Document S1. Figures S1–S6 and Tables S1 and S3–S6

Table S2. Splicing Silencer motifs detected in the AON biding region showed in Figure S1B Capital letters are representing the ABCA4 exon 39 base pairs.

Document S2. Article plus supplemental information
