# Supplementary material for: Antisense oligonucleotide therapy corrects splicing in the common Stargardt disease type 1-causing variant ABCA4 c.5461-10T>C
Source: Mol Ther Nucleic Acids. 2023 Feb 18;31:674–88. doi: 10.1016/j.omtn.2023.02.020 (PMC9999166; doi:10.1016/j.omtn.2023.02.020)
Supplement: Document S1. Figures S1–S6 and Tables S1 and S3–S6 [file mmc1.pdf]

OMTN, Volume *31*

Supplemental information

Antisense oligonucleotide therapy corrects splicing in the common Stargardt disease type 1-causing variant *ABCA4* c.5461-10T>C

Melita Kaltak, Petra de Bruijn, Davide Piccolo, Sang-Eun Lee, Kalyan Dulla, Thomas Hoogenboezem, Wouter Beumer, Andrew R. Webster, Rob W.J. Collin, Michael E. Cheetham, Gerard Platenburg, and Jim Swildens

Table S1. Sequences of antisense oligonucleotides used in the study.

AON Name	AON sequence
AON1	5'-CUCACAGGACAGCACAGGGCAA-3'
AON2	5'-CAGCAGGUGGGGCCCAGAUGCU-3'
AON3	5'-CAAACCCCACCCCCCCUCUCUU-3'
AON4	5'-GUAGGACUGUUGGAAACGGGG-3'
AON5	5'-AGCGUCUGAAACAGGGAA-3'
AON6	5'-GAACCUGAGCAGCGUCUGAAA-3'
AON7	5'-UGAGCAGCUUCCUCAGCACGG-3'
AON8	5'-CCCAGGCAGAAGUGGGGGAAG-3'
AON9	5'-GUGCAAGGUCAAUGAGGCCCC-3'
AON10	5'-CAAACCGGGCAUAGACAUCU-3'
AON11	5'-CUCGGCUACCACCCACCAAACC-3'
AON12	5'-CCCAGGGCCCAUGCUCCAUGGGC-3'
AON13	5'-GUAACCCUCCCAGCUUUGGA-3'
AON14	5'-GAGCCCCCCCGGUAACCCUCCCA-3'
AON15	5'-AGCACCAGCCCCUGCCACAGUC-3'
AON16	5'-UGCCACAGUCUGAUGCAGGAGCC-3'
AON17	5'-CCAGAUGCUCUCACAGGACAGCA-3'
AON18	5'-GGUGGGGCCCAGAUGCUCUCACA-3'
AON20	5'-CCCCUCUCUUCAGCAGGUGGGG-3'
AON21	5'-CCCACCCCCCCUCUCUUCAGCA-3'
AON23	5'-CAGGGAAGUAGGACUGUUGGAAAC-3'
AON24	5'-GCAGCGUCUGAAACAGGGAAGUAGG-3'
AON25	5'-GUUGAACCUGAGCAGCGUCUGAA-3'
AON26	5'-GGGAAGACAAUGAGCAGCUUCCU-3'
AON27	5'-AGGCCCCGGCCCAGGCAGAAGUG-3'
AON28	5'-GCCUGGCUCAGUGCAAGGUCAAU-3'
AON29	5'-ACCACCCACCAAACCGGGCAUAGA-3'
AON31	5'-UGCUCCAUGGGCCUCGGCUACCA-3'
AON32	5'-GGGCCCAUGCUCCAUGGGCCUCGG-3'
AON44	5'-AUGCUCCAUGGGCCUCGG-3'
AON59	5’-GCUCCAUGGGCCUCGG-3’
AON60	5’-UGCUCCAUGGGCCUCGG-3’

Table S2. The Excel file "Table S2" contains Splicing Silencer motifs detected in the AON binding region shown in Figure S1B. Capital letters are representing the *ABCA4* exon 39 sequence.

Table S3. Multiple unpaired t-test to identify significant differences in expression of genes between wild-type and homozygous c.5461-10T>C ROs at their age of 120 days, graphically displayed in Figure 3B.

	**Discovery?**	**P value**	**Mean of *ABCA4* c.5461-10T>C**	**Mean of Wild-type**	**Difference**	**SE of difference**	**t ratio**	**df**	**q value**
USH2A	No	0,191852	863,7	1048	-184,7	125,6	1,47	6	0,258361
CRX	No	0,167121	12759	8140	4619	2939	1,572	6	0,258361
NRL	No	0,346678	5772	3171	2601	2547	1,021	6	0,350145
NR2E3	No	0,633145	652,7	744,3	-91,63	182,3	0,5026	6	0,511582
ABCA4	Yes	0,000764	234,4	821	-586,7	93,56	6,27	6	0,003089

Table S4. *In silico* analysis of possible intergenic and intronic off-targets with 2 mismatches in QR-1011.

Gene	Matched bases	Distance to 5' exon	Distance to 3' exon
TEAD1	16/18	17 749	1 046
CARD19	16/18	-	9 737
MIPOL1	16/18	79 078	44 919
CAPN14	16/18	-	3 437
DDP4	16/18	4 033	25 850
TWIST2	16/18	35 943	25 182
ROBO2	16/18	369 388	9 934
PSD3	16/18	-	47 376
DLGAP2	16/18	304 389	46 477
GALNT9	16/18	7 285	36 930
CRAMP1	16/18	3 515	2 521
ATXN7L1	16/18	111 405	11 901
RNF111	16/18	-	8 938
GRIN2A	16/18	36 263	10 583
TTLL11	16/18	51 605	5 658
PPP1R26	16/18	-	225
FCGBP	16/18	695	780
LIMS1	16/18	4 115	22 717
PXYLP1	16/18	6 036	12 029
GET4	16/18	1 161	2 229
GRB2	16/18	5 195	55 539

Table S5. Raw data obtained after analysis of Western Blots with protein lysates shown in Figure 5B and Figure S6. The protein signal was relatively quantified using ImageJ image analysis software.

Lane	Sample	Protein mass (µg)	Signal (area)		Signal × protein mass		Mean WT signal		Factor		ABCA4/VCL
			ABCA4	VCL	ABCA4	VCL	ABCA4	VCL	ABCA4	VCL	
1	STGD1 untreated	85	0	16986.3	0	17985.5	40444.7	35140.4	0	0.5	0
2	STGD1 scrambled	85	0	22529	0	23854.2			0	0.7	0
3	STGD1 QR-1011	85	15010.6	32891	15893.6	34825.8			0.4	1	0.4
4	Wild-type	90	47863.2	39606.7	47863.2	39606.7			1.2	1.1	1.1
5	Wild-type	45	21372.8	17743.2	42745.5	35486.5			1.1	1	1.1
6	Wild-type	22.5	7681.4	7581.8	30725.6	30327.9			0.8	0.9	0.9

Lane	Sample	Protein mass (µg)	Signal (area)		Signal × protein mass		Mean WT signal		Factor		ABCA4/VCL
			ABCA4	VCL	ABCA4	VCL	ABCA4	VCL	ABCA4	VCL	
1	STGD1 untreated	85	0	8455.5	0	8952.9	28982.9	20984.8	0	0.4	0
2	STGD1 scrambled	85	0	10975.2	0	11620.8			0	0.6	0
3	STGD1 QR-1011	85	5462.5	17086.4	5783.8	18091.4			0.2	0.9	0.2
4	Wild-type	90	26324.3	18773.1	26324.3	18773.1			0.9	0.9	1
5	Wild-type	45	15236.5	9714.9	30473	19429.9			1.5	0.9	1.1
6	Wild-type	22.5	7537.9	6187.8	30151.5	24751.3			1	1.2	0.9

Lane	Sample	Protein mass (µg)	Signal (area)		Signal × protein mass		Mean WT signal		Factor		ABCA4/VCL
			ABCA4	VCL	ABCA4	VCL	ABCA4	VCL	ABCA4	VCL	
1	STGD1 untreated	85	0	7083.2	0	7499.9	31148.1	21941.4	0	0.3	0
2	STGD1 scrambled	85	0	11958.3	0	12661.7			0	0.6	0
3	STGD1 QR-1011	85	6895.1	14844	7300.6	15717.1			0.2	0.7	0.3
4	Wild-type	90	25998.6	15454.6	25998.6	15454.6			0.8	0.7	1.2
5	Wild-type	45	15142.9	11816.4	30285.8	23632.9			1	1.1	0.9
6	Wild-type	22.5	9290	6684.2	37159.8	26736.6			1.2	1.2	1

Table S6. Primer/probe list.

Target	Used for	Sequence (5'- 3')
ABCA4 intron 37	Midigene	GGTGGTGAATTCCCATGTGAACTGGCCAAGAG (FW)
ABCA4 intron 41	Midigene	TGCTTAGTCGACGAGAGCAAGGAGGGGAAGAG (RV)
RHO ex3 - *RHO* ex5 (*ABCA4* exon 39 skip)	Minigene	TACATGTTCGTGGTCCACTTC (FW)
		GCAGATGGTGGTGAGCAT (RV)
		/56-FAM/ACCGTCAAG/ZEN/GAGTTCCGGAACTG/3IABkFQ/
ABCA4 exon 39 inclusion	Minigene	TACATGTTCGTGGTCCACTTC (FW)
		GAAGACAATGAGCAGCTTCCT (RV)
		/56-FAM/AACGCTGCT/ZEN/CAGGTTCAACGC/3IABkFQ/
ABCA4 exons 39-40 skip	Midigene/ROs	GCGGTCATTCCCATGATGTA (FW)
		AACAATGGGCTCCTTAGTGG (RV)
		5HEX/AATAACCGG/ZEN/GATTGCCGAGCT/3IABkFQ
ABCA4 exon 39 skip	Midigene/ROs	GCGGTCATTCCCATGATGTA (FW)
		AACAATGGGCTCCTTAGTGG (RV)
		/56-FAM/AATAACCGG/ZEN/GTGAGGAGCAC/3IABkFQ/
ABCA4 wild-type (exons 39-40)	Midigene/ROs	GAGAATAACCGGACGCTGCT (FW)
		ACCACCATGGCAAACAGGTTC (RV)
		/56-FAM/CCCGGTTTG/ZEN/GTGAGGAGCAC/3IABkFQ/
OPN1MW	ROs	GCCACCCTATGTGTGTCC (FW)
		GCTGTCCACACAGCAGCC (RV)
		/5HEX/AGAAGGCAA/ZEN/TGCCCACGATGGC/3IABkFQ/
CRX	ROs	TTTGCCAAGACCCAGTACCC (FW)
		TTTAGCCCTCCGGTTCTTGA (RV)
		56-FAM/ATGCCCGTG/ZEN/AGGAGGTGGCT/3IABkFQ/
RHO	ROs	TCATGATGAACAAGCAGTTCC (FW)
		GTCTTGGACACGGTAGCAGA (RV)
		/5HEX/CATCTGCTGCGGCAAGAACCC/3IABkFQ/

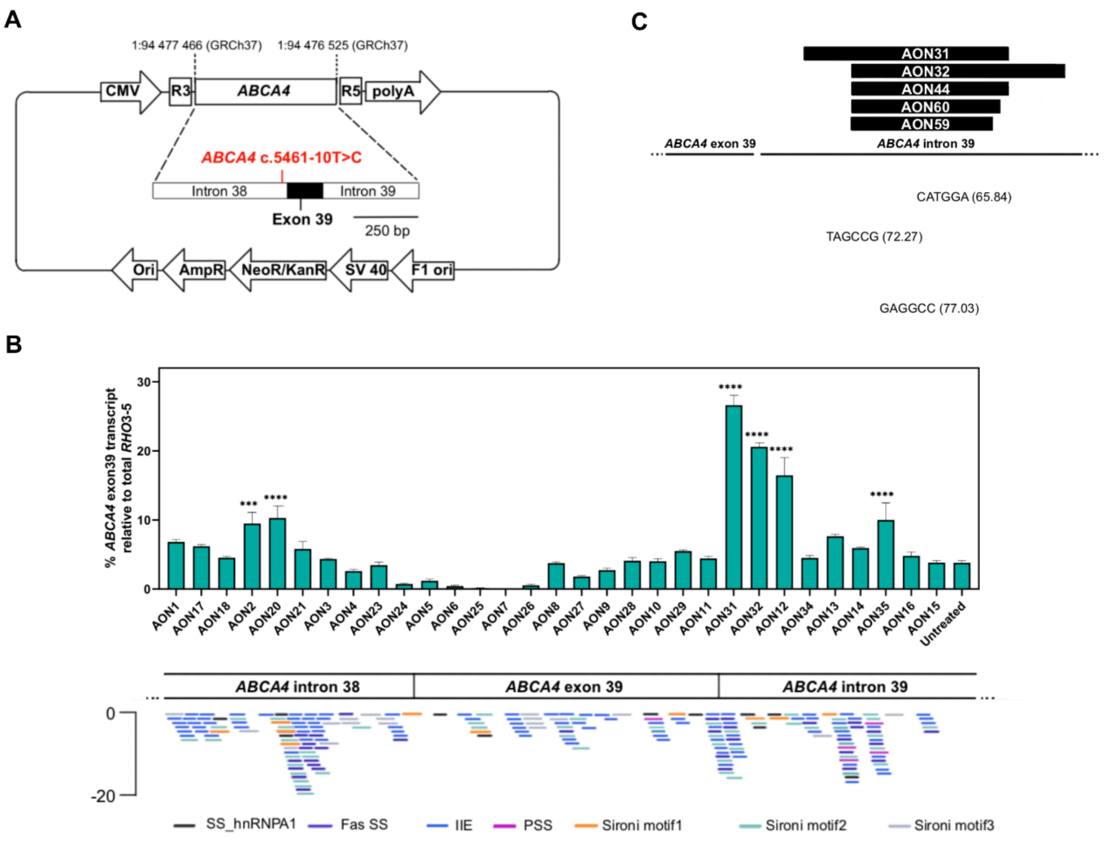

Figure S1. Screening in minigene-transfected HEK293 cells identifies target region with strong intronic splicing silencers. (A) Schematic representation of the minigene construct showing the *ABCA4* exon 39 flanked by parts of adjacent introns. (B) Splice rescue in the first AON screening on minigene-transfected cells. (n=2). Below are displayed the binding sites for RNA-splicing proteins (adapted from Human Splicing Finder).[1] ***$p \leq 0.001$, ****$p \leq 0.0001$. The details of each binding site can be found in Table S2. (C) Lead candidates AON31 and AON32 and their shorter versions AON44, AON60 and AON59 are complementary to the intron 39 region where three strong splicing silencer motifs are located that are involved in the recruitment of the splicing protein heterogeneous nuclear ribonucleoprotein A1 (hnRNP A1).

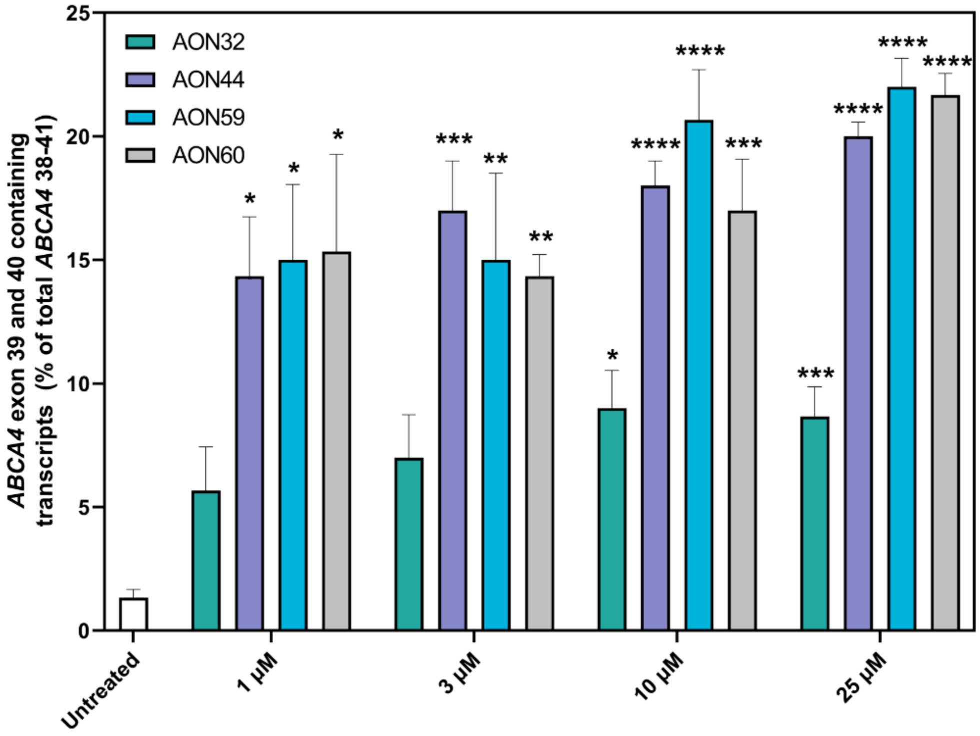

Figure S2. Shorter AONs are more effective. RNA analysis of midigene-transfected HEK293 cells treated gymnotically for 120 hours with best selected AONs. AON32 served as control since it consists in the long version of other selected. Data are shown as mean±s.e.m., n=3. All samples were compared to the untreated sample, *p≤0.05, **p≤0.01, ***p≤0.001, ****p≤0.0001.

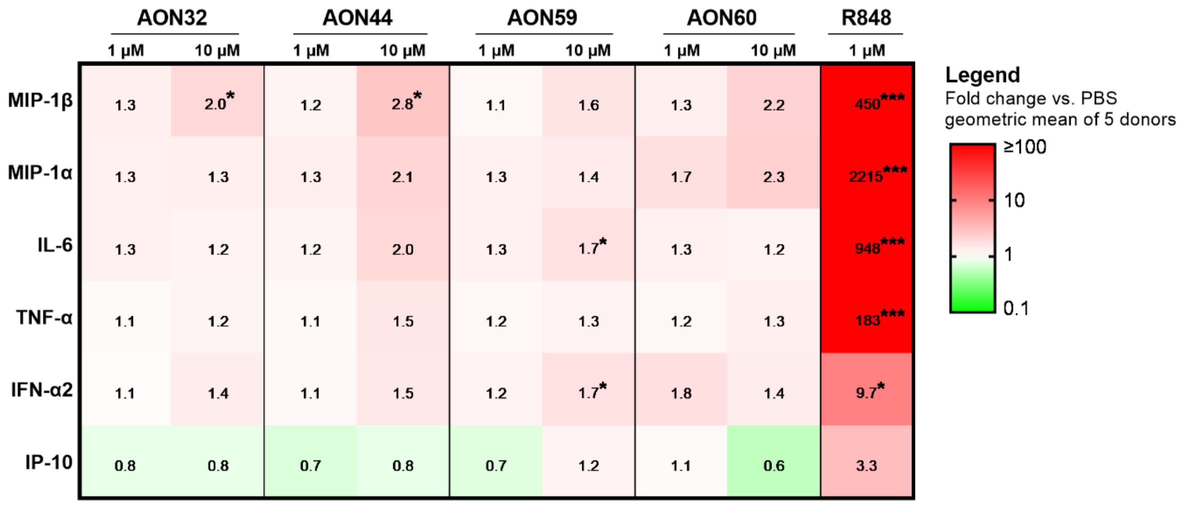

Figure S3. Immunostimulatory analysis of lead AON candidates reveals their slight influence on cytokine secretion. The heatmap depicts the fold change levels of cytokine concentrations in culture supernatant after 48-hour exposure to lead AON candidates or positive control R848 as compared to PBS-treated human peripheral blood mononuclear cells. Statistical testing was performed on log-transformed concentration values using mixed-model analysis, applying Dunnett's correction for multiplicity. Statistically significant differences vs. PBS were annotated with *$p<0.05$, **$p<0.01$, ***$p<0.001$.

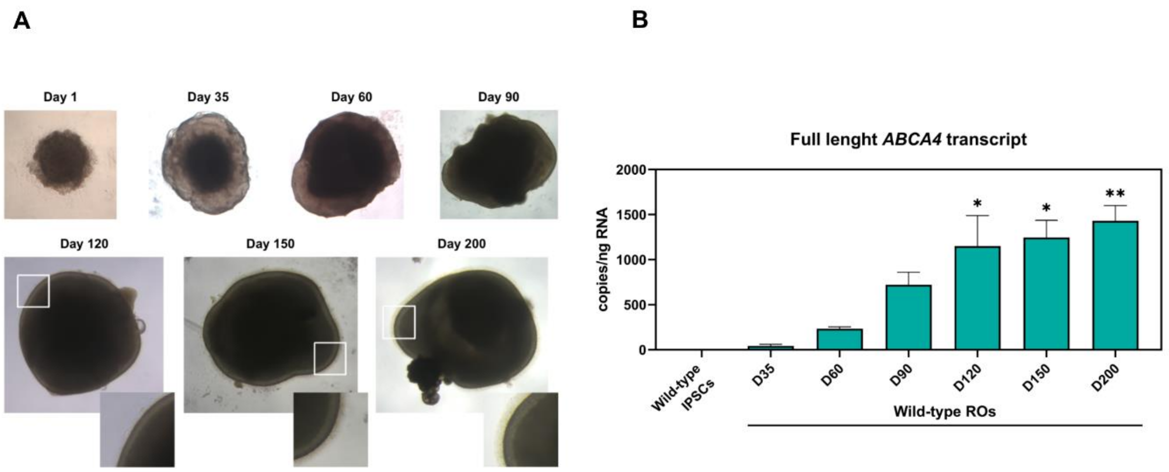

Figure S4. Morphological and *ABCA4* transcript analysis of wild-type ROs over time. (A) Control wild-type ROs were generated from control iPSCs that were the parent isogenic line for generation of gene edited homozygous *ABCA4* c.5461-10T>C iPSCs.[35] The iPSC appears as an embryoid body already at day 1 that develops into an organoid with dark core and sharp edges (day 35). The development of the neural retina was observed at day 90, and by day 120, the organoids were surrounded by a brush border that contains the inner and outer segments of photoreceptor cells. (B) The isoform analysis detected *ABCA4* transcripts only 35 days after organoid differentiation; the *ABCA4* expression increased at day 60 and 90 to 120, after which the expression is significantly higher over the expression detected in iPSCs. Moreover, 120 days after differentiation the expression of *ABCA4* remained relatively constant. Data are shown as mean±s.e.m., n=6. Statistically significant differences vs. wild-type iPSCs were annotated with $*p \leq 0.05$, $**p \leq 0.01$.

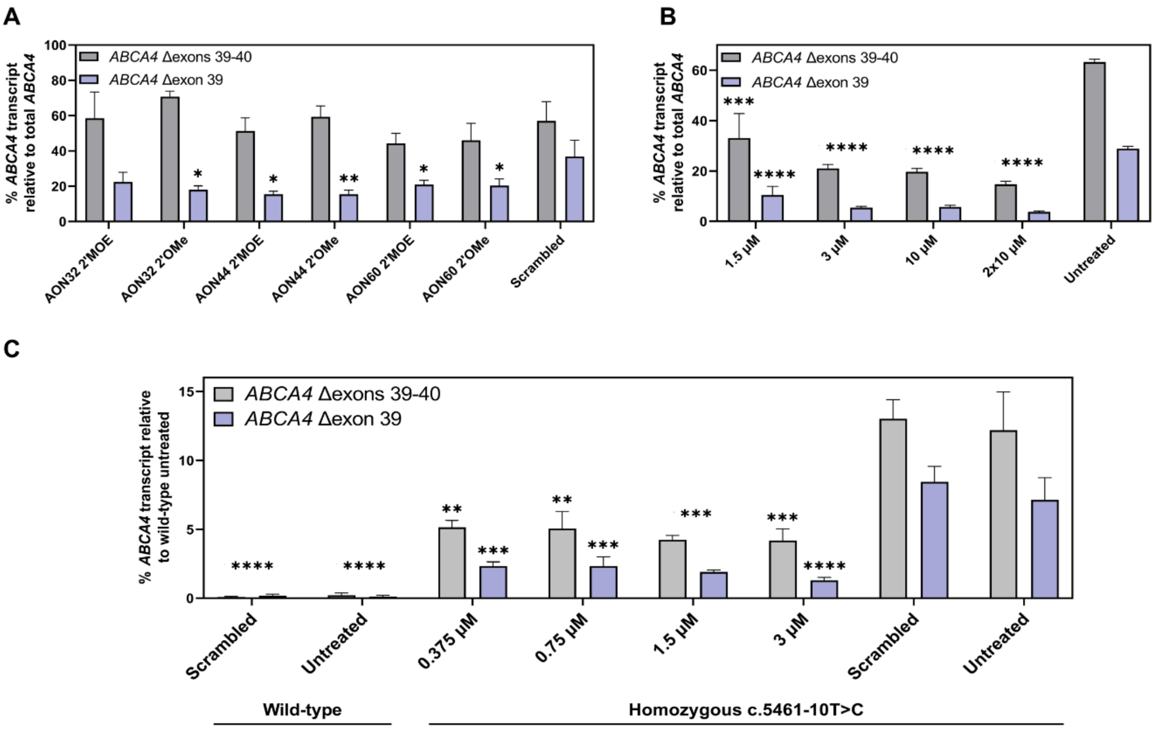

Figure S5 . AON treatments exhibit suppression of both truncated isoforms in ROs. (A) Percentages of single skip and double skip *ABCA4* isoforms in the organoid study described in Figure 4A, (B) Figure 4B and (C) Figure 5A. Data are shown as mean ± s.e.m. Statistically significant differences vs. untreated or scrambled were reported as *p≤0.05, **p≤0.01, ***p≤0.001, ****p≤0.0001, n=6.

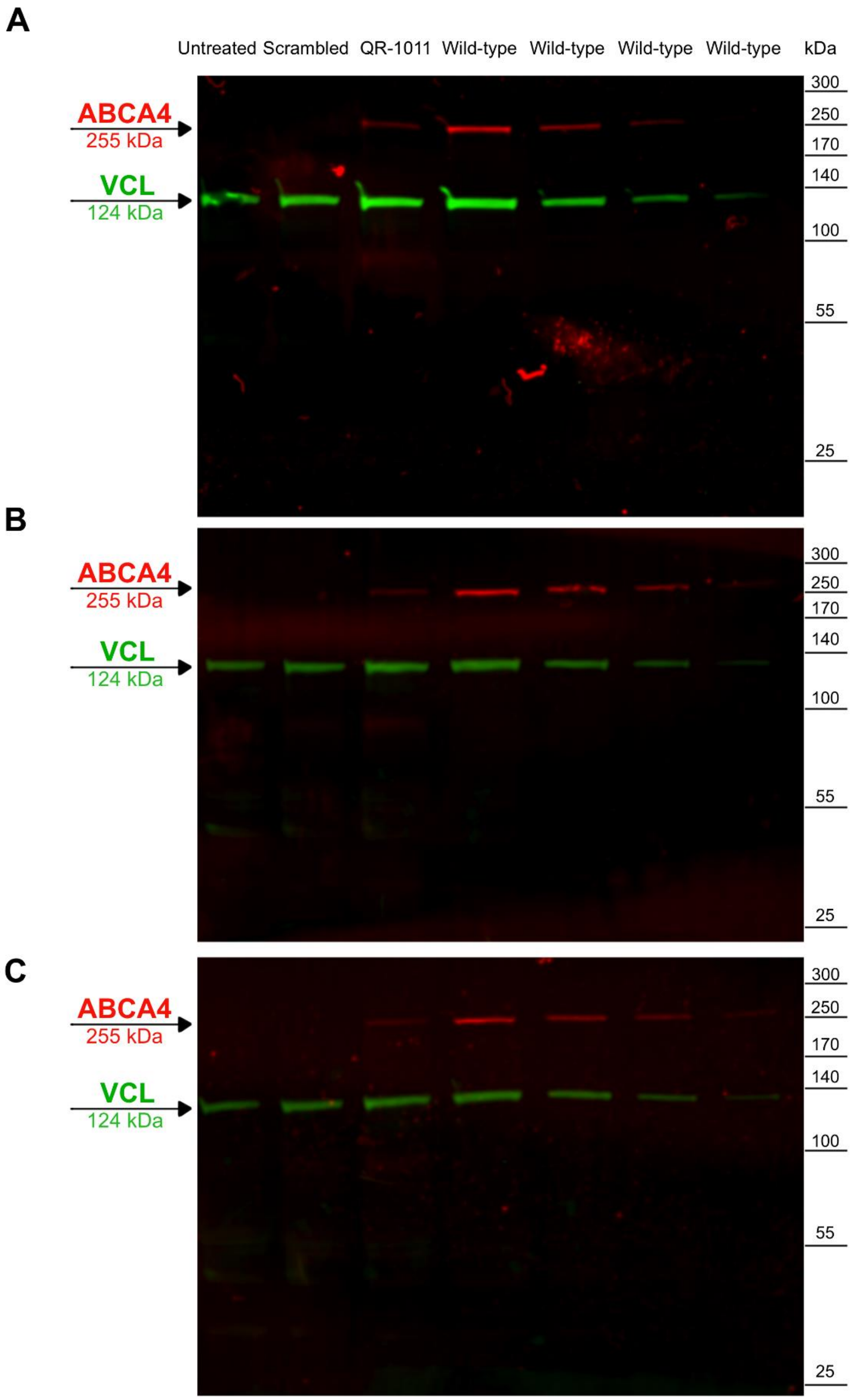

Figure S6. QR-1011 restores translation of wild-type ABCA4 in patient-derived ROs. Raw western blot data for the quantitative analysis (n=3) of protein lysates from patient-derived ROs treated with a 3 µM dose of QR-1011 and wild-type ROs (Figure 5B). Quantification details can be found in Table S5.

REFERENCES

1. Desmet, F.O., Hamroun, D., Lalande, M., Collod-Beroud, G., Claustres, M. and Beroud, C. (2009). Human Splicing Finder: an online bioinformatics tool to predict splicing signals. Nucleic Acids Res. *37*, e67. https://doi.org/10.1093/nar/gkp215.
